# Supplementary material for: Distance‐dependent distribution thresholding in probabilistic tractography
Source: Hum Brain Mapp. 2023 May 5;44(10):4064–76. doi: 10.1002/hbm.26330 (PMC10258532; doi:10.1002/hbm.26330)
Supplement: Supplementary file 1 — Data S1. Supporting information. [file HBM-44-4064-s001.docx]

**SUPPLEMENTARY MATERIALS**


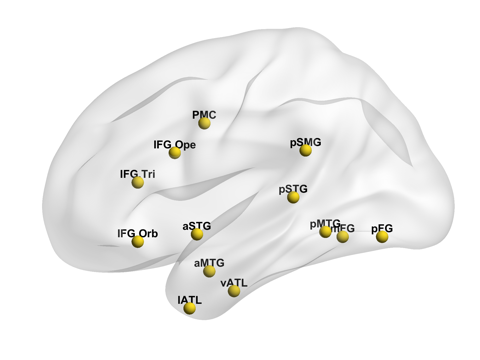


Figure S1. The projections of 13 language ROIs in standard space

.

Figure S2. The number of the ROI paired samples categorised into 15 distance ranges generated by using Euclidean distance, which is much less than the distance ranges reported in the main text.

Figure S3. The sampling distribution of random connectivity for each of the 15 distance ranges generated by using Euclidean distance. The x-axis indicates connection strength and the y-axis indicates the number of samples.

Figure S4. The distance-dependent distribution thresholds generated by using Euclidean distance at three alpha levels of 10%, 20% and 30% varied with the 15 ROI distance ranges.

Figure S5. The average language connectivity matrix generated by using Euclidean distance after thresholding based on the 15 distance ranges at the alpha levels of 10%, 20% and 30%. The connectivity matrix is very similar to Figure 6 reported in the main text. The dice similarity between the two matrices is 0.953.

Figure S6. The average language connectivity matrix across individuals after thresholding and binarization generated by using streamline distance. The different levels of alpha with 10% on the top, 20% on the middle, and 30% on the bottom were superimposed onto one matrix wherein anything that survives the 10% would also survive 20% and 30%.

Figure S7. The individual language connectivity matrices generated by using Euclidean distance for four representative participants after thresholding based on the 26 distance ranges at the alpha levels of 10%, 20% and 30%, as reported in the main text. Note that the colour scheme is hierarchical for simplification such that the green cells indicate thresholded connectivity in addition to the red cells, and the blue cells indicate thresholded connectivity in addition to both the red and green cells. As can be seen, there are individual differences in terms of brain connectivity and the strength of connectivity; however, the general pattern of connectivity is largely similar.

Figure S8. The dice similarity between the individual language connectivity matrices generated by using Euclidean distance and the average language connectivity matrix for all of the 54 individuals. As can be seen, the similarity scores are generally very high (M=0.883, SD=0.038). The result suggests that the DDD thresholds can also be directly applied to the individual level.
